# Extracting a COVID-19 signature from a multi-omic dataset

**DOI:** 10.3389/fbinf.2025.1645785

**Published:** 2025-09-22

**Authors:** Baptiste Bauvin, Thibaud Godon, Guillaume Bachelot, Claudia Carpentier, Riikka Huusaari, Maxime Deraspe, Juho Rousu, Caroline Quach, Jacques Corbeil

**Affiliations:** 1 GRAAL, Department d’Informatique et de Génie Logiciel, Université Laval, Québec, QC, Canada; 2 Corbeil Lab, Department of Molecular Medicine, CRCHU Université Laval, Québec, QC, Canada; 3 MILA, Quebec Artificial Intelligence Institute, Montreal, QC, Canada; 4 Linearis, Québec, Canada; 5 KEPACO, Department of Computer Science, Aalto University, Espoo, Finland; 6 Department of Microbiology, Infectious diseases and Immunology & Pediatrics, University of Montreal, Montreal, QC, Canada

**Keywords:** machine learning, multi-omics, biomarker, COVID-19, metabolomics, proteomics, signature

## Abstract

**Introduction:**

The complexity of COVID-19 requires approaches that extend beyond symptom-based descriptors. Multi-omic data, combining clinical, proteomic, and metabolomic information, offer a more detailed view of disease mechanisms and biomarker discovery.

**Methods:**

As part of a large-scale Quebec initiative, we collected extensive datasets from COVID-19 positive and negative patient samples. Using a multi-view machine learning framework with ensemble methods, we integrated thousands of features across clinical, proteomic, and metabolomic domains to classify COVID-19 status. We further applied a novel feature relevance methodology to identify condensed signatures.

**Results:**

Our models achieved a balanced accuracy of 89% ± 5% despite the high-dimensional nature of the data. Feature selection yielded 12- and 50-feature signatures that improved classification accuracy by at least 3% compared to the full feature set. These signatures were both accurate and interpretable.

**Discussion:**

This work demonstrates that multi-omic integration, combined with advanced machine learning, enables the extraction of robust COVID-19 signatures from complex datasets. The condensed biomarker sets provide a practical path toward improved diagnosis and precision medicine, representing a significant advancement in COVID-19 biomarker discovery.

## 1 Introduction

Coronavirus disease 2019 (COVID-19), caused by the severe acute respiratory syndrome coronavirus 2 (SARS-CoV-2), has led to the widespread deployment of diagnostic strategies, including reverse transcription-polymerase chain reaction (RT-PCR), antigen detection, and clinical symptom-based assessments. Clinically, the disease manifests with a heterogeneous spectrum of symptoms that can broadly be classified into three categories: infectious, respiratory, and neurological (Huang et al., 2020).

While RT-PCR detects viral RNA and antigen assays evaluate the humoral immune response, these tools alone do not capture the full complexity of the disease. COVID-19 has been associated with diverse systemic manifestations, including respiratory failure, coagulopathies, and inflammatory syndromes, some of which correlate with severe clinical outcomes and mortality risk (Iba et al., 2020; Lipman et al., 2022).

This phenotypic variability likely reflects a multifactorial disruption of underlying molecular and metabolic pathways. The resulting complexity underscores the need for integrative approaches to better understand host-pathogen interactions and to refine diagnostic and prognostic strategies.

Omics sciences, as key components of systems biology, offer powerful tools to address this challenge (Richard et al., 2022). By enabling high-throughput analysis of biological molecules such as genes, proteins, and metabolites, omics technologies provide a comprehensive view of the host response and disease progression. These approaches allow for the exploration of complex, multilayered mechanisms that underlie the clinical heterogeneity observed in COVID-19. However, omics approaches generate large amount of high-dimensional data, requiring advanced data analytics and machine learning methods to uncover meaningful biological insights.

The research dedicated to understanding COVID-19 has been extensive. Indeed, several studies have explored the application of machine learning to omics data in the context of COVID-19. However, many of these studies rely on a relatively limited number of biological markers, typically between 32 and a few hundred (Gong et al., 2022; Richard et al., 2022), which may not provide sufficient coverage for the discovery of novel biomarkers or for a comprehensive understanding of the underlying molecular mechanisms. From a computational perspective, most existing works employ single-model approaches (Liu et al., 2023) and do not systematically address class imbalance issues, which can impact model robustness and generalizability (Overmyer et al., 2020). In this paper, we aim to contribute to this body of knowledge by leveraging machine learning techniques to process the substantial amount of data collected during the pandemic. Our primary goal is to employ interpretable machine learning methodologies to identify a multi-omic signature for COVID-19 using data from a large cohort of patients. Machine learning models excel in uncovering complex multivariate relationships among features, unlike conventional statistical methods such as P-Value analysis, which typically focus on univariate correlations.

In addition to automated machine learning techniques, we integrated bioinformatics to analyze the proteomic and metabolomic data returned by the algorithms. We utilized the BQC19 database, a provincial initiative in Quebec designed to compile and analyze multiomic information to provide better insight into the COVID-19 pandemic. This study focuses specifically on proteomics and metabolomics data to ensure a substantial patient impact.

We present our machine learning pipeline for extracting signatures, offering both a high-level overview and detailed information about the models that form the foundation of our analysis. Our findings aim to improve the understanding of COVID-19 through advanced machine learning and bioinformatic approaches, ultimately forming better diagnostic and therapeutic strategies.

## 2 Materials and methods

### 2.1 Dataset

The data used in this paper was extracted from the Quebec COVID-19 biobank database^1^ (Tremblay et al., 2021), which provided data from 1400 COVID-19 positive and negative patients. In the original database, blood samples were processed to extract numerous -omic data, including genomic, proteomic, metabolomics, or transcriptomic. In our case, we chose to focus on metabolomic and proteomic data to run our workflow on biological information close to the targeted phenotype. Of all the available patient samples, we focused on patients who were hospitalized, either for COVID-19-related symptoms or for any other reason (scheduled interventions, consultations, among many other indications). These patients were sampled multiple times during their hospital stay, from which we only consider the first sampling. From this subgroup of patients, we extracted the subset that was available both in metabolomics and proteomics to enable multi-view approaches without the challenge of missing data. We only included original infections in the cohort and we focused on patients that were either positive for COVID-19 or negative but still presenting symptoms to ensure that our algorithms focused on COVID-19-specific biomarkers and not on the presence or absence of symptoms. Finally we selected patients that were sampled within 0–50 days following symptom onset. We thus obtained a cohort of 478 individuals, with 84% of COVID-19 positive patients and 16% of symptomatic controls, described by Table 1. Interestingly, this table shows that some symptoms, such as fever, output significant p-values to detect COVID-19-positive patients. However, we see in Section 3 that even if symptomatology provides some information, it was not up to the standard set by our method.

**TABLE 1:** Baseline characteristics. The p-value column indicates the result of a statistical test between the values of the variable and the variable COVID status.

		Grouped by status COVID				
		Missing	Overall	Positive	Control	P-value
n			478	375	103	
Sex:, n (%)	M	0	238 (49.8)	182 (48.5)	56 (54.4)	0.348
	F		240 (50.2)	193 (51.5)	47 (45.6)	
Age at arrival:, mean (SD)		0	66.0 (18.1)	66.4 (17.8)	64.6 (19.1)	0.389
Hospitalized or Outpatient? n (%)	Hosp	0	478 (100.0)	375 (100.0)	103 (100.0)	1.000
BMI:, mean (SD)		215	28.9 (6.5)	28.9 (6.2)	28.9 (7.4)	0.967
Days btwn sympt. and blood draw, mean (SD)		14	9.6 (8.4)	9.8 (7.8)	9.1 (10.5)	0.533
Obesity ? n (%)	No	1	432 (90.6)	337 (90.1)	95 (92.2)	0.643
	Yes		45 (9.4)	37 (9.9)	8 (7.8)	
Asymptomatic? n (%)	No	0	454 (95.0)	363 (96.8)	91 (88.3)	0.001
	Yes		24 (5.0)	12 (3.2)	12 (11.7)	
Shortness of breath (Dyspnea) ? n (%)	No	175	27 (8.9)	16 (6.6)	11 (17.7)	0.013
	Yes		276 (91.1)	225 (93.4)	51 (82.3)	
Fever ( > 38.0 Celcius) ? n (%)	No	197	40 (14.2)	19 (7.8)	21 (55.3)	< 0.001
	Yes		241 (85.8)	224 (92.2)	17 (44.7)	
Muscle aches (Myalgia) ? n (%)	No	354	69 (55.6)	46 (47.9)	23 (82.1)	0.003
	Yes		55 (44.4)	50 (52.1)	5 (17.9)	
Loss of taste/lost of smell ? n (%)	No	375	79 (76.7)	55 (69.6)	24 (100.0)	0.005
	Yes		24 (23.3)	24 (30.4)	0 (0)	

For each patient, 5,400 targeted metabolites were tested. The data acquisition method is detailed in the work of Tremblay et al. (2021), including the handling of batch effects and data normalization. As our main goal was to identify biomarkers, we focused on the subset of 896 metabolites that were identified by either a KEGG (Kanehisa and Goto, 2000) or an HMDB (Wishart et al., 2007) index to ensure our ability to track their associated metabolic pathways without any ambiguity. In Supplementary Material, we provide experiments run on the 5,400 available metabolites, quantifying the loss in accuracy induced by this metabolite filtering. Moreover, from the 7,200 tested proteins, we used the 5,284 identified proteins available in the dataset and discarded the un-identified ones. Therefore, our approaches relied on two dataset, a metabolomic one of size 478 × 896 and a proteomic one of size 478 × 5284, with no missing data. We provide the code that retraces the pre-processing pipeline in the GitHub repository.

### 2.2 Extracting signatures form multi-omic data

The main goal of our study was to extract a small subset of features, a signature, strongly associated with the phenotype from the multi-omic data provided by BQC-19. To select the best features for the COVID-19 signature, we trained interpretable machine learning algorithms to differentiate COVID-19 positive and negative patients from which we extracted and ranked the most important features.

Additionally, to compare our approach to state-of-the-art analysis, we performed a statistical study on all the available features both in metabolomics and proteomics. It implied constructing a volcano plot, showing differences in average values of data features between the positive and negative folds. The volcano plot reports the fold change (ratio between the two average values in folds) in the x-axis and the p-value of a statistical T-test that shows the significance of differences between the average values in the two folds on the y-axis. We used this statistical analysis to build a subset of features that were significant both in terms of fold change (a ratio lower than 0.5 or higher than 2) and p-value (p-value <0.05/6180 ). Bonferroni correction is applied to the threshold on the p-value (standard threshold of 0.05 divided by the number of variables tested. This selection process yielded a subset of 10 features, which we refer to as the Volcano signature. This subset serves as a comparative baseline for evaluating the efficacy of our machine learning-based feature selection approach.

#### 2.2.1 Interpretable classifiers

When applying machine learning to sensitive tasks such as health-related problems, it is highly recommended to use models that are understandable by non-machine learning experts (Rudin, 2019). Machine learning is a vast field in which models vary from tremendously complex deep neural networks (Nielsen, 2015) to straightforward logic-based Set Covering Machines (Marchand and Shawe-Taylor, 2003). Our study focused on algorithms that present one characteristic: natively outputting an importance score for each feature. Indeed, understanding the models outputted by more complex classifiers is a research field in itself (Arrieta et al., 2020), but in our case, we use only classifiers that output inherently interpretable models as Explainable AI methods are commonly less precise than native feature importances (Wang et al., 2024).

In this work, we are interested in two outputs for each classifier: first, the quality of their model, measured by its classification accuracy. Secondly, the importance score they assign to each feature of the dataset, which indicates the significance of that feature in the decision-making process, denoted feature importance. It is important to note that our work does not assume these models are sufficiently reliable to autonomously classify COVID-19 patients. Instead, they serve as a tool to extract significant features from the dataset. This set of features is then validated through bioinformatics analysis, as described in Section 2.5, and could contribute to a better understanding of long COVID-19 symptomatology.

#### 2.2.2 Feature relevance

**FIGURE 1:**
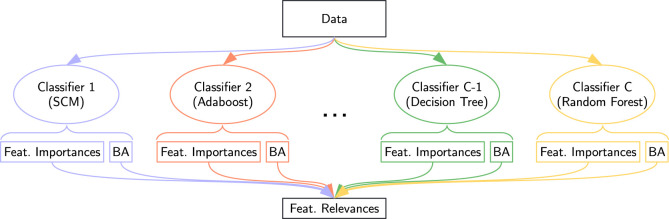
Feature relevance extraction: Models from various families are fitted to the data, each providing a balanced accuracy (BA) score reflecting its performance and a feature importance score indicating the contribution of each feature. Feature relevance is the weighted average of feature importance across all models, where weights are determined by each model’s balanced accuracy.

One notable contribution of this study is the aggregation of classifier outputs to compute a single score for each feature, quantifying its general utility for the COVID-19 classification task. Therefore, we introduced the concept of feature relevancecomputed as the average feature importance across classifiers, weighted by each classifier’s quality. This relevance score reflects a consensus among classifiers, with each classifier “voting” based on the feature’s importance in its model, weighted by the model’s overall performance, as illustrated in Figure 1. Moreover, a feature deemed important in low-accuracy classifiers is less relevant than one moderately important in high-accuracy models. It is similar to the method proposed in Fisher et al. (2019), diverging in the fact that we built a majority vote taking into account all the available classifiers where Fisher et al. (2019) sets an accuracy threshold under which the classifiers are not considered. In Definition 1, we provide a mathematical formulation for the relevance.

DEFINITION 1 (Feature relevance). Considering a benchmark, comprised of C classifiers $h_{1},\ldots ,h_{c},\ldots ,h_{C}$ . Each one outputting a classification score $s_{c}$ , and a feature importance score $F_{c,j}$ for each feature j of the dataset, we define the relevance of a feature $r_{j}$ as the average of the feature importance weighted by the score of each classifier as in Equation 1.

$$r_{j}=\overset{C}{\underset{c=1}{\sum }}n\left(s_{c}\right)\times F_{c,j},$$ (1)

with $n\left(s_{k}\right)$ the normalized score computed, for a higher-is-better scoring method as in Equation 2

$$n\left(s_{c}\right)=\left\{\begin{array}{cc} 0\; & \text{ if }s_{c}<r,\\ \frac{s_{c}-r}{m-r}\; & \text{otherwise,} \end{array}\right.$$ (2)

with r the score of the random guessing classifier and m the maximum score.

#### 2.2.3 Applicative challenges

The dataset in this study is significantly imbalanced, with 375 COVID-19 positive patients and 103 controls. To address this, we employed the balanced accuracy metric (Brodersen et al., 2010), which averages the accuracy of each class, ensuring a model predicting all patients as positive only achieves 50% balanced accuracy.

**FIGURE 2:**
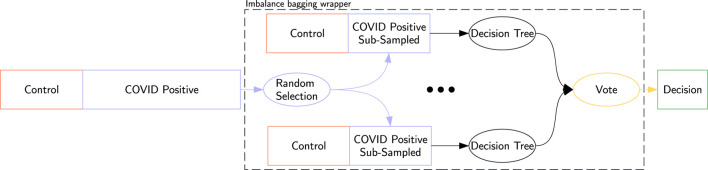
Imbalance bagging for a decision tree: Random sub-sampling creates 10 balanced datasets. Each dataset trains a classifier, and the final decision is the aggregate vote of these classifiers, denoted IB Decision Tree.

Additionally, we applied an imbalance bagging (IB) wrapper (Lemaître et al., 2017) to compatible classifiers. This sampling strategy was selected for its ability to reduce variance and improve robustness when used with tree-based classifiers, as well as to avoid introducing synthetic or unrealistic observations that could distort the original data distribution. This method runs 10 classifiers on re-balanced subsets of the dataset, where the number of COVID-19-positive samples matches the number of control samples. Each IB classifier aggregates the votes from these 10 sub-classifiers. An example using a decision tree is shown in Figure 2.

The dataset offers multi-omic data for signature extraction but poses a challenge with its “fat” structure, where the number of features (6,180) far exceeds the number of samples (487) (Romero et al., 2017). This contrasts with “big” data, where the samples outnumber features. Learning on fat data is a challenge frequently encountered when applying machine learning to biological datasets. To address this, we used sparse classifiers with built-in dimension reduction and conducted experiments across 10 train/test splits to enhance robustness, a process we term train/test bootstrapping.

### 2.3 Full experimental protocol

**FIGURE 3:**
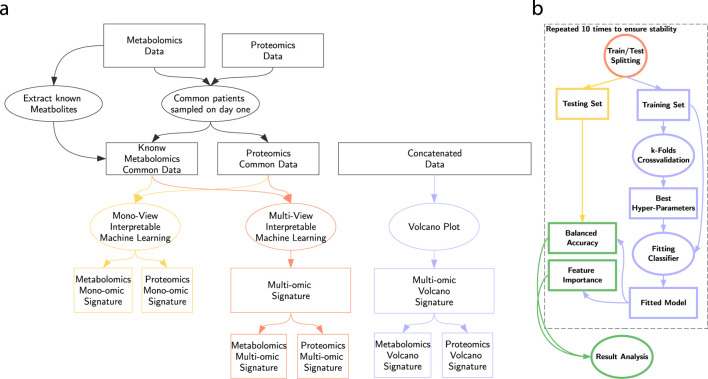
Study workflow. **(a)** Signature extraction process. **(b)** ML workflow.

In this section, we detail our protocol, summarized in Figure 3. We first separated the data into two views with no missing data: metabolomics and proteomics. In addition, we trained our approaches on their concatenation (Snoek et al., 2005), denoted the multi-omic view. On each of those versions of the dataset, we run our process to extract a metabolomic, proteomic and multi-omic signature, as presented in Figure 3a.

The machine learning workflow represented in Figure 3b consists of splitting the dataset 10 times in a training (80%) and a testing (20%) set, maintaining the original class ratio. On each split, we trained a pool of classifiers described in depth in Section 2.4. We optimized hyper-parameters via random search combined with 5-fold cross-validation. Finally, we evaluate their balanced accuracy on the testing set and we combine the feature importance scores of each train/test split.

All the machine learning experiments were run on SuMMIT (Bauvin et al., 2022), facilitating comparisons between mono- and multi-omic classifiers.

### 2.4 In-depth machine learning algorithms

#### 2.4.1 Interpretable classifiers pool

To provide feature relevance for each classifier, we focused on interpretable classifiers. Ensemble methods were primarily used due to their capability on -omic data and inherent interpretability when using straightforward sub-classifiers.

Among ensemble methods, we focused on sparse approaches, such as the Set Covering Machine (SCM) (Marchand and Shawe-Taylor, 2003; Drouin et al., 2016), which builds a logical combinations of binary rules. We then included Decision Tree (DT) (Breiman et al., 1984), a well-known method used for its interpretability, despite a higher risk of overfitting.

For more complex decision functions, Adaboost (Freund and Schapire, 1997) and Gradient Boosting (Friedman, 2001) were included. They perfectly fit our study, as, with our parameters, they rely on single-feature sub-classifiers that are linearly combined. We also used SamBA (Bauvin et al., 2023), which integrates both the advantages of boosting and similarity-based decisions for precise and sparse functions. Random Forest (Breiman, 2001) and RandomSCM (Godon et al., 2022) were utilized despite their generally dense decision functions, owing to the feature relevance calculation’s capability to manage dense classifiers.

We also included kernel methods, known for their versatility with complex tasks (Hofmann et al., 2008). Traditional kernel methods (e.g., SVM-RBF) often lack interpretability due to complex decision functions. We addressed this with SPKM (Huusari et al., 2021), which introduces primal and dual sparsity, making kernel-based models interpretable by relying on sparse elements.

#### 2.4.2 Additional state-of-the-art classifiers

For performance analysis of extracted signatures, less interpretable algorithms were included. Lasso (Tibshirani, 1996) was added to assess the linear separability of patients. k-Nearest Neighbors (KNN) (Fix and Hodges, 1989) and Support Vector Machine with Radial Basis Function kernel (SVM-RBF) (Boser et al., 1992) served as indicators of the signal-to-noise ratio in the signatures. Note that SVM-RBF’s sensitivity to imbalance led to the inclusion of its IB variant. Post-extraction, these algorithms provided insights into the biological processes underlying the signatures.

### 2.5 Pathway enrichment analysis, visualization, and interpretation

#### 2.5.1 Proteomics mono-view signature

We interpreted the proteomics mono-view signature using a two-step approach. First, we performed pathway enrichment analysis (PEA) using the ConsensusPathDB (CPDB) web tool to identify key regulatory mechanisms and signaling pathways of SARS-CoV-2. CPDB integrates data from 32 public resources, including KEGG, Reactome, and WikiPathways. We used an over-representation statistical method to identify enriched biological functions in the list of significant proteins, with a p-value cutoff of 0.01 and false discovery rate (FDR) correction. A custom proteome reference background was uploaded to reduce false positives. Second, we conducted a pathway topology-based analysis using NetworkAnalyst software to highlight physical and functional interactions among genes and proteins. We used the InnateDB database to retrieve binary interactions and visualized high-confidence relationships as a network. Functional pathway analysis was performed on seed proteins, and the top Gene Ontology Biological Process (GO:BP) terms and biochemical pathways were selected for visualization in Cytoscape. This approach combines over-representation analysis with network-based analysis to provide a comprehensive interpretation of the proteomics signature.

#### 2.5.2 Metabolomic mono-view signature

The interpretation of the metabolomic mono-view signature relied on pathway over-representation analysis (ORA) to study the inflammatory immune response and metabolic perturbations in SARS-CoV-2 patients. We standardized the list of significant metabolite identifiers using the Metabolite ID Conversion tool in MetaboAnalyst 5.0 and built a custom metabolome reference background. ORA and pathway topology (PT) analyses were performed on the metabolic mono-view signature (Top17) and a broader signature (Top50) using the MetPA module in MetaboAnalyst 5.0. Default parameters were used, including the KEGG human pathway library, hypergeometric test for ORA, and relative betweenness centrality for PT, with FDR correction to minimize false positives. The STITCH web tool was used to explore interactions between proteins and metabolites, providing insights into molecular and cellular functional relationships. STITCH links chemical space to gene-protein space through physical and functional interactions stored in the STRING database. The STITCH web tool was used to explore interactions between proteins and metabolites, providing insights into molecular and cellular functional relationships. STITCH links chemical space to gene-protein space through physical and functional interactions stored in the STRING database. A network association for the broader mono-view signature (Top50) was performed to maximize metabolite-metabolite interactions (min interaction score of 0.4 and up to 20 first-order interactors) and retrieving biochemical reactions from the STRING database and PubChem text mining. Functional enrichments of GO biological processes and KEGG pathways were performed and imported into Cytoscape for interpretation of the metabolomic signature.

## 3 Results

### 3.1 Classifier performance on full description

**FIGURE 4:**
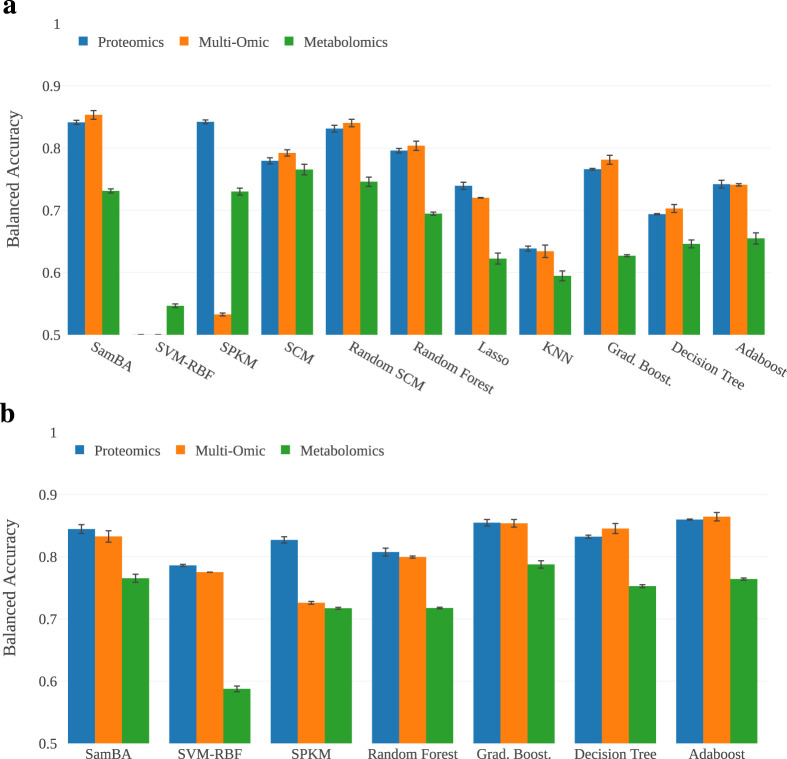
Performance of the algorithms on the full description. The majority of the approaches are over 70% balanced accuracy. **(a)** Basic algorithms scores on the full description of the samples. Each view is represented by a coloured bar. The best performing algorithm is SamBA on the multi-omic data. **(b)** Scores for the algorithms for which we added the imbalanced bagging wrapper presented in Section 2.2.3. The best performing algorithm is Adaboost on the multi-omic dataset.

The first stage of this study is to evaluate the classifiers on the full dataset, analyzing their scores on metabolomic, proteomic and multi-omic data. In Figure 4a, we provide a bar plot representing the mean balanced accuracies (BA) for each classifier on each representation of the data. In Figure 4b, we report the BA of the IB derivatives of the classifiers that were sensitive to imbalance. In this experiment, the scores range from 87% BA for the IB version of Adaboost on proteomics to 50% BA for the SVM-RBF on multi-omics and proteomics. From these figures, we can infer that for the task at hand, proteomics provides more information on the COVID-19 status of the patients than metabolomics. However, metabolomics is still informative as a classifier relying uniquely on it outputs 79% balanced accuracy.

### 3.2 Signature extraction

**FIGURE 5:**
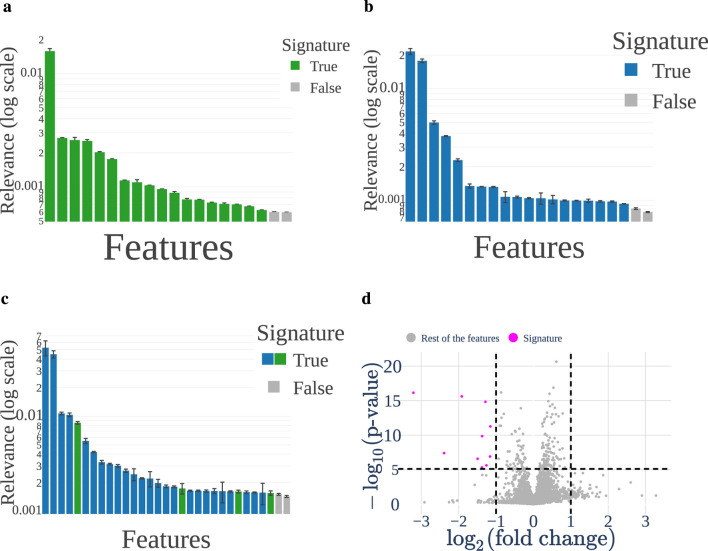
**(a–c)** show the relevance ranking for the features in metabolomics, proteomics and multi-omic, respectively. The blue features represent the signatures. In Supplementary Material 1.2, we report all the features ranked by relevance for multi-omics, highlighting the small size of the signatures compared to the full descriptions. **(d)** Shows the volcano plot with one point being a feature represented by its fold change (x) and p-value (y). All the features that are in the top right and left corners are selected as part of the Volcano signature. **(a)** Metabolomics. **(b)** Proteomics. **(c)** Multi-omic. **(d)** Volcano plot.

Relying on these encouraging results, we computed the feature relevance for each descriptor of both the -omics, and obtained three graphs, presented in Figures 5a–c, one for each -omic and one for the multi-omics. In these graphs, we plotted the relevance of each feature, as introduced in Section 2.2.2. We highlight the features included in the signatures in blue, and the other ones in orange. Therefore,

• for metabolomics, we set the relevance threshold to $6.5*10^{-4}$ ,

• for proteomics to $9*10^{-4}$ ,

• for multi-omics to $1.51*10^{-3}$ .

This provides us with a 17 feature signature for metabolomics, 19 for proteomics and 29 for multi-omics. The list of bio-markers included in our signatures is provided in Tables 2–4. In addition to those relevance-based signatures, our study uses one additional subset of features: the significant features outputted by the volcano plot shown in Figure 5d, denoted the Volcano signature.

**TABLE 2:** Proteomics signature.

Feature	Relevance
MX1	2.16 $e^{-02}$
ISG15	1.78 $e^{-02}$
LAG3	4.98 $e^{-03}$
IFIT3	3.76 $e^{-03}$
TNXB	2.29 $e^{-03}$
C1QTNF1	1.34 $e^{-03}$
CXCL10	1.32 $e^{-03}$
EPHB2	1.32 $e^{-03}$
SCGB3A1	1.07 $e^{-03}$
F11	1.07 $e^{-03}$
LUM	1.05 $e^{-03}$
SDCBP	1.04 $e^{-03}$
PRDM1	1.02 $e^{-03}$
KRT7	9.96 $e^{-04}$
INSR	9.93 $e^{-04}$
DDX58	9.92 $e^{-04}$
APCS	9.78 $e^{-04}$
NMI	9.74 $e^{-04}$
HRASLS2	9.31 $e^{-04}$

**TABLE 3:** Metabolomics signature.

Feature	Relevance
Ribothymidine	1.58 $e^{-02}$
Ureidopropionic acid	2.70 $e^{-03}$
PC(34:4)	2.59 $e^{-03}$
N-Acetylputrescine	2.55 $e^{-03}$
PC(P-16:0/16:1 (9Z))	2.02 $e^{-03}$
Cytosine	1.76 $e^{-03}$
Salicyluric acid	1.14 $e^{-03}$
2-Ketobutyric acid	1.10 $e^{-03}$
Succinimide	1.03 $e^{-03}$
Alpha-Ketooctanoic acid	9.56 $e^{-04}$
Malic acid	8.87 $e^{-04}$
PC(18:2 (9Z,12Z)/18:2 (9Z,12Z))	7.74 $e^{-04}$
Cer(d18:1/16:0)	7.70 $e^{-04}$
Azithromycin	7.26 $e^{-04}$
Oleamide	7.07 $e^{-04}$
Kynurenine	7.00 $e^{-04}$
2-Furoylglycine	6.73 $e^{-04}$

**TABLE 4:** Multi-omic signature.

Omic	Feature	Relevance
P	MX1	5.27 $e^{-02}$
P	ISG15	4.50 $e^{-02}$
P	IFIT3	1.07 $e^{-02}$
P	LAG3	1.04 $e^{-02}$
M	Ribothymidine	8.54 $e^{-03}$
P	TNXB	5.51 $e^{-03}$
P	CXCL10	4.21 $e^{-03}$
P	F11	3.29 $e^{-03}$
P	EPHB2	3.15 $e^{-03}$
P	KRT7	3.01 $e^{-03}$
P	APCS	2.68 $e^{-03}$
P	ADPGK	2.45 $e^{-03}$
P	LGALS9	2.23 $e^{-03}$
P	C1GALT1C1	2.21 $e^{-03}$
P	BGLAP	1.98 $e^{-03}$
P	C1QTNF1	1.84 $e^{-03}$
P	INSR	1.81 $e^{-03}$
M	LysoPE (20:4 (5Z,8Z,11Z,14Z)/0:0)	1.74 $e^{-03}$
P	DDX58	1.65 $e^{-03}$
P	LUM	1.65 $e^{-03}$
P	STAT1	1.64 $e^{-03}$
P	PREP	1.64 $e^{-03}$
P	ADSL	1.63 $e^{-03}$
P	HRASLS2	1.62 $e^{-03}$
M	(4-ethenyl-2-methoxyphenyl)oxidanesulfonic acid	1.62 $e^{-03}$
P	IL1R1	1.59 $e^{-03}$
P	HEXB	1.58 $e^{-03}$
P	MUCL1	1.57 $e^{-03}$
M	Methyllysine	1.56 $e^{-03}$

### 3.3 Performance scores on signatures

**FIGURE 6:**
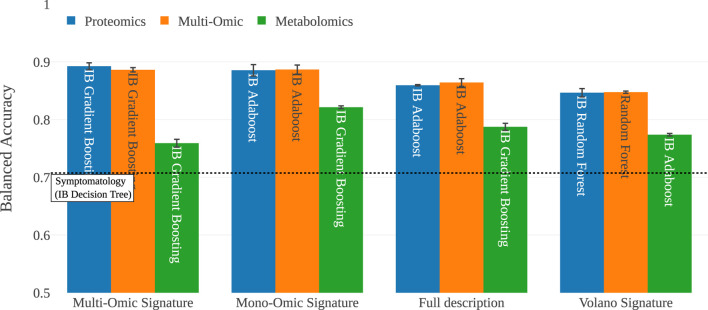
This figure shows the score of the best performing algorithm for each signature on each view. The full description encapsulates all the features available, and the symptomatology baseline, represented as a dashed line, shows a classifier’s performance relying only on symptomatology data, as shown in Table 1.

Relying on those subsets of features, we analyze them by re-training the pool of classifiers on each subset. In Figure 6, for the sake of clarity, we only present the score of the best algorithm on each subset of features. In Supplementary Material, we provide the matrix that gathers the accuracy of all the classifiers on all the signatures.

Figure 6 shows that the best scores are obtained on the multi-omic signature, despite the fact that it consists of only 29 features among the 6,180 available ones. This result confirms that the proposed multi-omic signature is relevant to separate symptomatic patients between COVID-19 and controls. Another interesting result is the low accuracy scores of the volcano signature. Indeed, machine learning presents the huge advantage of including interactions between features in its models, which is not the case with univariate statistical analyses such as volcano plots or p-Values (Costanzo et al., 2022). Therefore, even if the Volcano plot extracts a 21-feature signature, its accuracy is never greater than 84% . In the following section, we investigate the pathways linked to each of the extracted features to further ensure that the machine learning study yielded meaningful features.

### 3.4 Pathway enrichment analysis and network-based analysis

#### 3.4.1 Proteomic mono-view signature

A machine learning-based proteomic analysis identified 19 differentially expressed proteins (DEPs) in COVID-19 positive patients compared to negative ones. The most predictive biomarkers were MX1, ISG15, LAG3, IFIT3, and TNXB. Pathway enrichment analysis revealed that these DEPs were significantly associated with SARS-CoV-2 innate immunity evasion and cell-specific immune response pathways. Notably, interferon-related pathways, such as ISG15 antiviral mechanism and interferon signaling, were highly enriched. Proteins MX1, ISG15, CXCL10, and DDX58 were the most frequently mapped in COVID-19-related pathways. Functional interactions among these biomarkers will be further evaluated through protein network analysis. Additionally, some pathways were linked to other viral infections like influenza A and hepatitis C, indicating a broader disturbance in the innate immune response. Proteins such as EPHB2, APCS, and F11 were associated with disease severity, including coagulopathies and lung damage. A protein-protein interaction network analysis highlighted ISG15 as a key protein, interacting with MX1, IFIT3, and DDX58. These proteins were enriched in interferon-related pathways and immune responses to viruses. The analysis also identified interferon regulatory factors (IRF-1, IRF-4, IRF-7) and STAT1 as major mediators of the cellular response to interferons. Overall, the proteomic signature is highly correlated with interferon signaling following viral infection (Menachery and Gralinski, 2021; Buchrieser et al., 2020; Lin et al., 2021; Rajah et al., 2021).

#### 3.4.2 Metabolomic mono-view signature

Metabolomics identified 12 differentially expressed metabolites and 5 lipids that segregate SARS-CoV-2 patients, highlighting the role of lipid metabolism in inflammatory and immune responses. Ribothymidine showed the most significant variation, previously reported as a COVID-19 biomarker. Azithromycin, a broad-spectrum antibiotic, was upregulated in COVID-19 positive patients, potentially due to its administration during the pandemic. Pathway analysis did not reveal significant enrichment in the KEGG database, but top biomarkers were associated with pyrimidine metabolism. Key metabolites included ureidopropionic acid, α -ketobutyric acid, and L-malic acid, which were mapped across multiple interconnected pathways. Network analysis identified five main sub-clusters of metabolites, with L-Kynurenine (KYN) as a key metabolite in the tryptophan and kynurenine cluster. Enzymes involved in the KYN pathway, such as indoleamine 2,3-dioxygenase (IDO5) and kynurenine aminotransferase (AADAT and CCBL26), were also identified. Polyamine metabolites were found to favor coronavirus replication, and spermidine was highlighted in the polyamine sub-cluster. The metabolization of niacinamide to 1-methyl nicotinamide, important for the innate immune response, was also noted. Overall, the metabolomic signature is associated with amino acids, carbohydrates, nucleotides, and lipids, with significant enrichments in nicotinate, nicotinamide, beta-alanine, arginine, proline, and tryptophan metabolisms.

## 4 Discussion

COVID-19 emerged as a global health crisis in early 2020, characterized by complex pathophysiology, diverse clinical manifestations, and the persistence of symptoms in a subset of patients, known as long COVID. The aim of this study was to gain deeper insight into the intricate molecular mechanisms involved in the disease.

Characterizing the biological response to COVID-19 remains a major challenge. This study underscores the added value of exploring multiple omic layers to obtain a more comprehensive view of the underlying pathophysiology. Despite leveraging a relatively large cohort from the BQC-19 dataset, the sample size remains limited when compared to the complexity of biological systems and the depth of omics-level interactions. Additionally, the limited overlap between patients with full multi-omics profiles precluded the use of robust, interpretable multi-omic machine learning models.

This study focuses on extracting signatures relying on machine learning approaches. Such methods present the huge advantage of taking into consideration both linear and non-linear relations between the data and the outcome. By combining multiple algorithms, we mathematically approach the problem from different perspectives, enabling the identification of diverse and potentially complementary signal patterns (capture of both linear and non-linear relationships). Indeed, machine learning algorithms are able to find very complex patterns in the data, compared to classical statistical analysis (Costanzo et al., 2022). This advantage comes with a potentially much higher computational cost. However, we only used approaches that were compatible with very high-dimensional data to reduce the computational needs of our pipeline. In addition, machine learning models, by their very nature, set hypotheses based on the shape of the patterns they try to learn. To overcome this, we aggregated a large collection of classifiers thanks to our feature relevance score.

The reduced signature retrained on a smaller subset of features proved particularly robust, as it helped minimize noise from weakly informative variables and improve generalization.

Finally, in Section 3.2, we propose signatures that are solely based on the relevance of the features, setting thresholds that take into account breaking points in the relevance rankings. To complete such a method, an ablation study (Cohen and Howe, 1988) or another more advanced explainability method could be complementary to our interpretable approach. This collaboration between interpretability and explainability could allow us to optimize the number of features in each signature without relying on an arbitrary threshold.

Although initially designed as an exploratory and mechanistic approach, the results and models developed in this study could pave the way for novel biomarkers or diagnostic signatures that go beyond the binary outcomes provided by antigen or PCR tests, thereby enabling more precise therapeutic strategies based on the involved metabolic axis (Yamga et al., 2023) However, this approach poses several challenges, particularly the need for rigorous, multi-center, prospective validation, as well as careful selection of the variables to be included in the clinical score, while accounting for analytical constraints.

Several of the top-ranked markers identified in our results are highly consistent with the known pathophysiology of COVID-19, notably involving viral infection responses and immune hyperactivation, which are often responsible for severe symptoms or even death. The presence of multiple interferon-stimulated genes such as MX1, ISG15, and IFIT3 reflects a robust antiviral response, while proteins like CXCL10, LAG3, and STAT1 further support the role of immune dysregulation and hyperinflammation in COVID-19 progression. The presence of coagulation factor XI (F11) is also notable, potentially reduced in COVID-19 cases, echoing its implication in hemorrhagic and thrombotic complications observed in severe disease. Markers such as C1QTNF1 and APCS additionally point to disturbances in vascular and complement pathways, which are frequently associated with endothelial damage and systemic inflammation. Additionally, the detection of azithromycin among the discriminating features likely reflects its widespread therapeutic use in COVID-19 patients, though it is not a biologically meaningful marker *per se*, as it is not directly linked to underlying pathophysiological mechanisms.

Taken together, our findings emphasize the interplay of immune and inflammatory responses, coagulation and vascular dysfunction, and metabolic disturbances, all of which align with the known molecular and clinical features of COVID-19.

Interestingly, a greater proportion of proteomic markers emerged in the multi-omic signature, corroborating the superior predictive performance observed with proteomics-based models. This suggests that the proteomic layer captures more informative biological signals in the context of COVID-19, likely reflecting the protein-driven nature of immune activation and inflammatory cascades during infection.

Nonetheless, the metabolomic signature contributes distinct and complementary insights, highlighting alterations in lipid metabolism (e.g., phosphatidylcholines, ceramides), amino acid catabolism (e.g., kynurenine, N-acetylputrescine), and energy pathways (e.g., malic acid, 2-ketobutyric acid). These findings reflect broader metabolic adaptations and stress responses that are not fully captured at the proteomic level. By integrating both omics layers, the multi-omic model offers a more comprehensive and mechanistic view of the host response, bridging upstream immune signaling and downstream metabolic consequences. This systems-level perspective may help explain the heterogeneity of clinical outcomes in COVID-19 and could contribute to the development of more nuanced diagnostic or prognostic tools.

In addition, our current study only relies on correlation relation, which could be improved by working with causal approaches once they are up to the standards of our pipeline (Godon et al., 2023). In addition, this work is one of the first steps in the discovery of a signature for long COVID-19.

This study has limitations. It was conducted on a single patient cohort with both metabolomic and proteomic data available. An interesting extension would be to test mono-omic signatures on patients with data from only one omics type. However, the imbalance in case-control numbers would require specific methodological adjustments. Moreover, the control group’s heterogeneity may introduce bias—especially in relation to symptom onset kinetics and associated molecular signatures. Still, given the urgency and complexity of COVID-19 during the pandemic, our design enabled the identification of relevant omic signatures in line with existing literature and prevailing biological hypotheses.

Lastly, this study represents an early step toward identifying molecular signatures associated with long COVID. Future efforts involving larger, longitudinal, and multi-omic cohorts will be essential to fully characterize long-term post-infection consequences.

## Data Availability

The data analyzed in this study is subject to the following licenses/restrictions: Available on request at https://en.quebeccovidbiobank.ca/. Requests to access these datasets should be directed to https://en.quebeccovidbiobank.ca/soumission-d-une-demande-d-acc%C3%A8s.

## Footnotes

1
https://www.bqc19.ca/

## References

[B1] Arrieta A. B. Díaz-Rodríguez N. Del Ser J. Bennetot A. Tabik S. Barbado A. (2020). Explainable artificial intelligence (xai): concepts, taxonomies, opportunities and challenges toward responsible ai. Inf. Fusion 58, 82–115. 10.1016/j.inffus.2019.12.012

[B2] Bauvin B. Corbeil J. Benielli D. Koço S. Capponi C. (2022). “Integrating and reporting full multi-view supervised learning experiments using summit,” in Proceedings of the fourth international workshop on learning with imbalanced domains: theory and applications, 139–150.

[B3] Bauvin B. Capponi C. Clerc F. Germain P. Koço S. Corbeil J. (2023). “Sample boosting Algorithm (SamBA) - an interpretable greedy ensemble classifier based on local expertise for fat data,” in Proceedings of the thirty-ninth conference on uncertainty in artificial intelligence. Editors Evans R. J. Shpitser I. (Pittsburgh, PA: JMLR) 216, 130–140. Available online at: https://proceedings.mlr.press/v216/bauvin23a.html .

[B4] Boser B. E. Guyon I. M. Vapnik V. N. (1992). “A training algorithm for optimal margin classifiers,” in Proceedings of the fifth annual workshop on computational learning theory, 144–152. 10.1145/130385.130401

[B5] Breiman L. (2001). Random forests. Mach. Learn. 45, 5–32. 10.1023/A:1010933404324

[B6] Breiman L. Friedman J. H. Olshen R. A. Stone C. J. (1984). Classification and regression trees. Wadsworth and Brooks.

[B7] Brodersen K. H. Ong C. S. Stephan K. E. Buhmann J. M. (2010). “The balanced accuracy and its posterior distribution,” in 2010 20th International Conference on Pattern Recognition, Istanbul, Turkey, 23-26 August 2010, 3121–3124. 10.1109/ICPR.2010.764

[B8] Buchrieser J. Dufloo J. Hubert M. Monel B. Planas D. Rajah M. M. (2020). Syncytia formation by SARS-CoV-2-infected cells. EMBO J. 39, e106267. 10.15252/embj.2020106267 33051876 PMC7646020

[B9] Cohen P. R. Howe A. E. (1988). How evaluation guides ai research: the message still counts more than the medium. AI Mag. 9, 35.

[B10] Costanzo M. Caterino M. Fedele R. Cevenini A. Pontillo M. Barra L. (2022). Covidomics: the proteomic and metabolomic signatures of covid-19. Int. J. Mol. Sci. 23, 2414. 10.3390/ijms23052414 35269564 PMC8910221

[B11] Drouin A. Giguère S. Déraspe M. Marchand M. Tyers M. Loo V. G. (2016). Predictive computational phenotyping and biomarker discovery using reference-free genome comparisons. BMC Genomics 17, 754. 10.1186/s12864-016-2889-6 27671088 PMC5037627

[B12] Fisher A. Rudin C. Dominici F. (2019). All models are wrong, but many are useful: learning a variable’s importance by studying an entire class of prediction models simultaneously. J. Mach. Learn. Res. 20, 177–181. 34335110 PMC8323609

[B13] Fix E. Hodges J. L. (1989). Discriminatory analysis. Nonparametric discrimination: consistency properties. Int. Stat. Rev. 57, 238–247. 10.2307/1403797

[B14] Freund Y. Schapire R. E. (1997). A decision-theoretic generalization of on-line learning and an application to boosting. Jour. Comp. Sys. Sci. 55, 119–139. 10.1006/jcss.1997.1504

[B15] Friedman J. H. (2001). Greedy function approximation: a gradient boosting machine. Ann. Statistics 29, 1189–1232. 10.1214/aos/1013203451

[B16] Godon T. Plante P.-L. Bauvin B. Francovic-Fontaine É. Drouin A. Corbeil J. (2022). Randomscm: interpretable ensembles of sparse classifiers tailored for omics data. ArXiv abs/2208.06436.

[B17] Godon T. Bauvin B. Germain P. Corbeil J. Drouin A. (2023). Invariant causal set covering machines. ArXiv abs/2306.04777.

[B18] Gong H. Wang M. Zhang H. Elahe M. F. Jin M. (2022). An explainable ai approach for the rapid diagnosis of covid-19 using ensemble learning algorithms. Front. Public Health 10, 874455. 10.3389/fpubh.2022.874455 35801239 PMC9253566

[B19] Hofmann T. Schölkopf B. Smola A. J. (2008). Kernel methods in machine learning. Ann. Statistics 36, 1171–1220. 10.1214/009053607000000677

[B20] Huang C. Wang Y. Li X. Ren L. Zhao J. Hu Y. (2020). Clinical features of patients infected with 2019 novel coronavirus in wuhan, China. Lancet 395, 497–506. 10.1016/S0140-6736(20)30183-5 31986264 PMC7159299

[B21] Huusari R. Bhadra S. Capponi C. Kadri H. Rousu J. (2021). Learning primal-dual sparse kernel machines. CoRR abs/2108.12199.

[B22] Iba T. Levy J. H. Levi M. Thachil J. (2020). Coagulopathy in covid-19. J. Thrombosis Haemostasis 18, 2103–2109. 10.1111/jth.14975 32558075 PMC7323352

[B23] Kanehisa M. Goto S. (2000). KEGG: kyoto encyclopedia of genes and genomes. Nucleic Acids Res. 28, 27–30. 10.1093/nar/28.1.27 10592173 PMC102409

[B24] Lemaître G. Nogueira F. Aridas C. K. (2017). Imbalanced-learn: a python toolbox to tackle the curse of imbalanced datasets in machine learning. J. Mach. Learn. Res. 18, 1–5.

[B25] Lin L. Li Q. Wang Y. Shi Y. (2021). Syncytia formation during SARS-CoV-2 lung infection: a disastrous Unity to eliminate lymphocytes. Cell Death Differ. 28, 2019–2021. 10.1038/s41418-021-00795-y 33981020 PMC8114657

[B26] Lipman D. M. Safo S. E. Chekouo T. (2022). Multi-omic analysis reveals enriched pathways associated with covid-19 and covid-19 severity. PLoS ONE 17, e0267047–30. 10.1371/journal.pone.0267047 35468151 PMC9038205

[B27] Liu X. Hasan M. R. Ahmed K. A. Hossain M. Z. (2023). Machine learning to analyse omic-data for covid-19 diagnosis and prognosis. BMC Bioinforma. 24, 7. 10.1186/s12859-022-05127-6 36609221 PMC9817417

[B28] Marchand M. Shawe-Taylor J. (2003). The set covering machine. J. Mach. Learn. Res. 3, 723–746.

[B29] Menachery V. D. Gralinski L. E. (2021). Coagulation and wound repair during COVID-19. J. Heart Lung Transpl. 40, 1076–1081. 10.1016/j.healun.2021.06.006 34334300 PMC8195688

[B30] Nielsen M. A. (2015). Neural networks and deep learning, vol. 25. San Francisco, CA, USA: Determination press.

[B31] Overmyer K. A. Shishkova E. Miller I. J. Balnis J. Bernstein M. N. Peters-Clarke T. M. (2020). Large-scale multi-omic analysis of covid-19 severity. Cell Syst. 12, 23–40.e7. 10.1016/j.cels.2020.10.003 33096026 PMC7543711

[B32] Rajah M. M. Bernier A. Buchrieser J. Schwartz O. (2021). The mechanism and consequences of sars-cov-2 spike-mediated fusion and syncytia formation. J. Mol. Biol. 434, 167280. 10.1016/j.jmb.2021.167280 34606831 PMC8485708

[B33] Richard V. R. Gaither C. Popp R. Chaplygina D. Brzhozovskiy A. Kononikhin A. (2022). Early prediction of covid-19 patient survival by targeted plasma multi-omics and machine learning. Mol. Cell. Proteomics 21, 100277. 10.1016/j.mcpro.2022.100277 35931319 PMC9345792

[B34] Romero A. Carrier P. L. Erraqabi A. Sylvain T. Auvolat A. Dejoie E. (2017). “Diet networks: thin parameters for fat genomics,” in 5th International Conference on Learning Representations, ICLR 2017, Toulon, France, April 24-26, 2017.

[B35] Rudin C. (2019). Stop explaining Black box machine learning models for high stakes decisions and use interpretable models instead. Nat. Mach. Intell. 1, 206–215. 10.1038/s42256-019-0048-x 35603010 PMC9122117

[B36] Snoek C. Worring M. Smeulders A. (2005). Early versus late fusion in semantic video analysis. Proc. 13th ACM Int. Conf. Multimedia 2005, 399–402doi. 10.1145/1101149.1101236

[B37] Tibshirani R. (1996). Regression shrinkage and selection via the lasso. J. R. Stat. Soc. Ser. B Methodol. 58, 267–288. 10.1111/j.2517-6161.1996.tb02080.x

[B38] Tremblay K. Rousseau S. Zawati M. H. Auld D. Chassé M. Coderre D. (2021). The Biobanque québécoise de la COVID-19 (BQC19)—A cohort to prospectively study the clinical and biological determinants of COVID-19 clinical trajectories. PLoS One 16, e0245031. 10.1371/journal.pone.0245031 34010280 PMC8133500

[B39] Wang H. Liang Q. Hancock J. Khoshgoftaar T. (2024). Feature selection strategies: a comparative analysis of shap-value and importance-based methods. J. Big Data 11, 44. 10.1186/s40537-024-00905-w

[B40] Wishart D. S. Tzur D. Knox C. Eisner R. Guo A. C. Young N. (2007). HMDB: the human metabolome database. Nucleic Acids Res. 35, D521–D526. 10.1093/nar/gkl923 17202168 PMC1899095

[B41] Yamga E. Soulé A. Piché A. Emad A. Durand M. Rousseau S. (2023). Validation of ANG-1 and P-SEL as biomarkers of post-COVID-19 conditions using data from the Biobanque québécoise de la COVID-19 (BQC-19). Clin. Proteomics 20, 44. 10.1186/s12014-023-09436-7 37875801 PMC10594676

