# Supplementary material for: Extracting a COVID-19 signature from a multi-omic dataset
Source: Front Bioinform. 2025 Sep 22;5:1645785. doi: 10.3389/fbinf.2025.1645785 (PMC12497780; doi:10.3389/fbinf.2025.1645785)
Supplement: Supplementary file 1 [file DataSheet1.pdf]

Supplementary Material

1 ADDITIONAL RESULTS

1.1 All metabolites comparison

Figure S1 shows the discrepancy in balanced accuracy between the models that learned on all the available metabolites of the BQC-19, and the models that learned only on metabolites that were identified by either a KEGG or HMDB index.

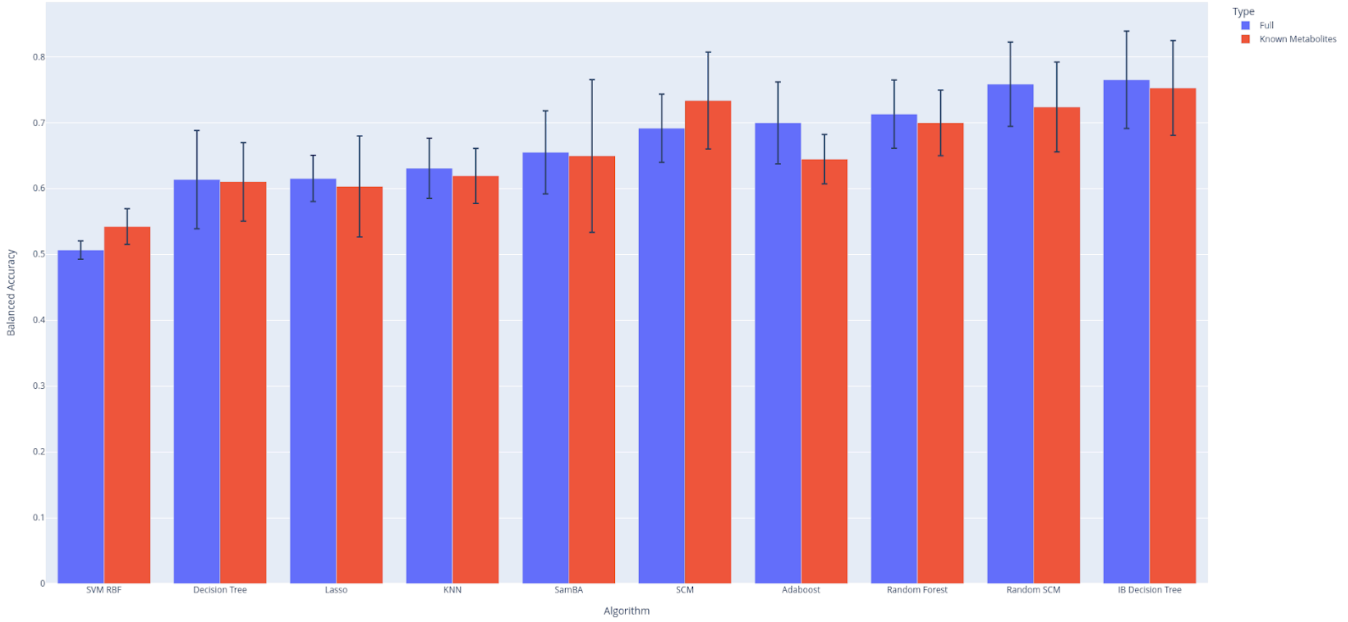

Figure S1. Comparison of the balanced accuracy of our pool of classifiers on the entire set of available metabolites (Full) and the ones provided with either a KEGG or HMDB index (Known)

The full description is slightly more informative but, given the standard deviation, we consider that using only the known metabolites is not too deleterious for the metabolomics view.

1.2 Feature relevance ranking

Figure S2 displays the relevance ranking of all the features that are non-zero.

1.3 Full numerical results - Balanced accuracy

Figure S3 reports all the balanced accuracies of the models that were fitted on the available data.

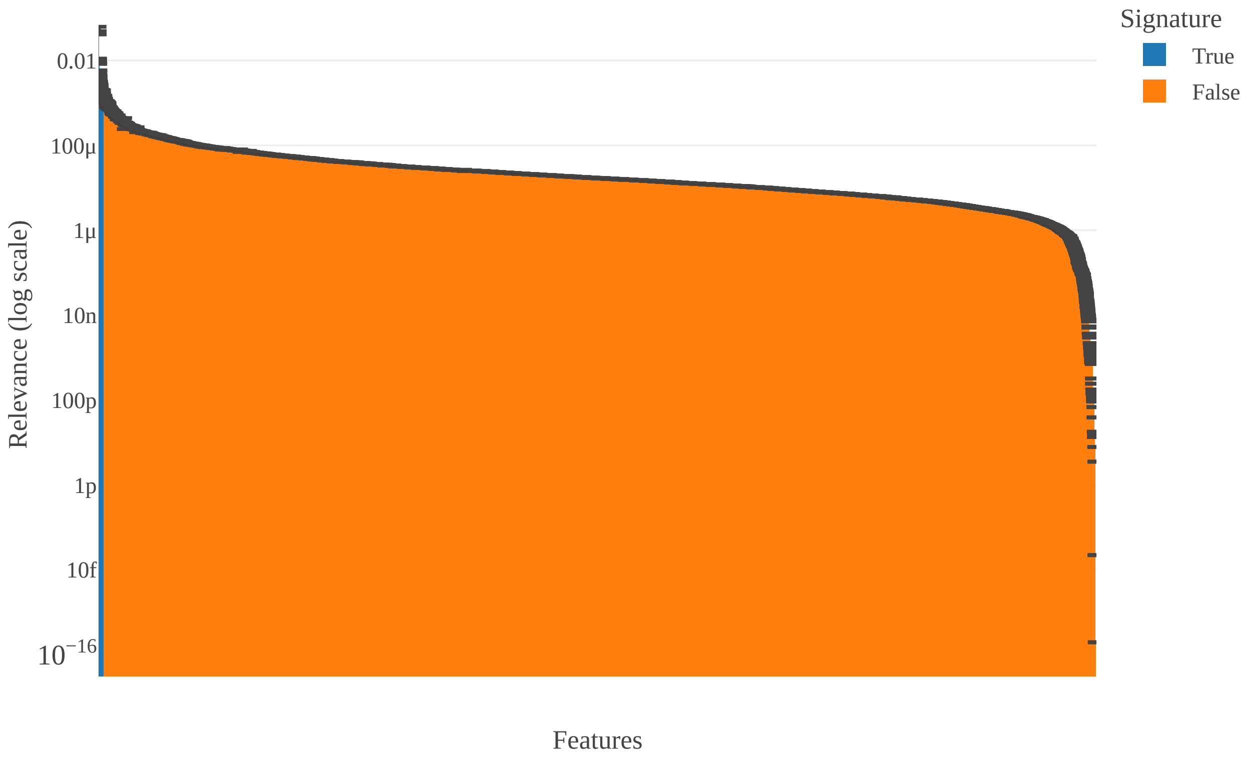

Figure S2. Feature relevance ranking for all non-zero features. The signature is plotted in blue, and the discarded features in orange.

	Decision Tree	IB Decision Tree	SCM	Adaboost	IB Adaboost	Grad. Boost.	IB Gradient Boosting	Random SCM	Random Forest	IB Random Forest	SamBA	IB SamBA	SPKM	IB SPKM	Lasso	SVM-RBF	IB SVM-RBF	KNN	Mean
Mean	69.81	80.39	76.62	72.96	82.44	73.35	83.14	79.49	80.14	81.26	81.01	80.98	70.35	71.02	64.26	52.26	73.12	64.38	74.28
Symptomatology	63.49 ± 6.85	70.74 ± 3.87	66.76 ± 6.67	60.82 ± 4.69	66.31 ± 7.45	60.18 ± 4.31	70.05 ± 4.15	66.61 ± 8.06	68.98 ± 2.57	69.56 ± 3.41	68.94 ± 2.88	69.8 ± 3.78	58.91 ± 4.41	58.62 ± 7.11	55.26 ± 4.97	50.0 ± 0.0	62.31 ± 5.16	53.98 ± 2.74	63.41
Multi-omic signature	74.67 ± 7.37	85.11 ± 6.62	81.39 ± 4.57	81.51 ± 4.49	88.47 ± 4.02	81.17 ± 4.18	88.64 ± 3.37	85.66 ± 6.13	86.27 ± 4.83	86.72 ± 3.07	86.3 ± 4.56	85.3 ± 4.4	83.06 ± 3.94	85.08 ± 3.55	77.28 ± 4.21	50.45 ± 0.9	81.41 ± 3.79	73.3 ± 3.23	81.21
Both mono-omics signatures	72.97 ± 4.97	84.13 ± 5.43	81.01 ± 4.44	79.31 ± 6.37	88.68 ± 3.79	79.52 ± 5.28	87.93 ± 4.46	86.7 ± 5.25	84.92 ± 5.74	87.3 ± 4.09	86.29 ± 4.3	85.07 ± 4.5	66.5 ± 5.47	54.89 ± 3.43	69.9 ± 6.06	54.37 ± 3.74	81.53 ± 4.03	70.8 ± 3.19	77.88
Multi-omic Union (volcano & signature)	75.47 ± 5.75	82.54 ± 6.38	80.25 ± 5.84	75.63 ± 4.96	86.62 ± 5.13	78.97 ± 7.37	87.65 ± 3.9	83.97 ± 3.7	84.51 ± 5.12	84.59 ± 5.05	86.39 ± 4.62	85.4 ± 5.0	63.31 ± 2.56	73.91 ± 5.28	67.34 ± 5.81	52.35 ± 1.54	80.55 ± 3.88	65.7 ± 7.13	77.51
Multi-omic volcano signature	70.46 ± 4.91	81.73 ± 6.0	79.13 ± 4.86	75.0 ± 5.36	83.65 ± 4.87	75.86 ± 5.99	83.2 ± 4.76	82.74 ± 4.83	84.75 ± 4.2	84.07 ± 3.89	83.11 ± 3.16	81.06 ± 5.86	64.73 ± 4.84	58.8 ± 6.47	60.69 ± 3.84	52.05 ± 1.7	79.3 ± 2.82	66.84 ± 4.81	74.84
Full multi-omic data	70.28 ± 5.64	84.52 ± 4.8	79.21 ± 6.5	74.09 ± 5.19	86.43 ± 6.67	78.11 ± 5.73	85.36 ± 6.61	84.02 ± 3.6	80.36 ± 6.74	79.93 ± 4.17	85.32 ± 4.7	83.25 ± 4.91	53.25 ± 3.23	72.58 ± 7.22	72.0 ± 6.05	50.0 ± 0.0	77.51 ± 5.01	63.41 ± 3.99	75.54
Proteomic multi-omic signature	75.5 ± 5.49	86.68 ± 3.89	80.66 ± 5.44	82.41 ± 4.57	87.86 ± 4.63	78.15 ± 5.7	89.25 ± 4.59	85.22 ± 6.42	86.56 ± 5.28	87.12 ± 3.2	85.92 ± 4.6	85.87 ± 4.22	84.73 ± 4.95	85.34 ± 4.87	76.32 ± 5.16	50.31 ± 1.21	81.48 ± 3.59	74.19 ± 6.68	81.31
Metabolomic multi-omic signature	61.67 ± 6.59	72.16 ± 2.77	71.34 ± 6.11	64.11 ± 4.72	73.46 ± 7.19	65.45 ± 6.65	75.91 ± 4.68	72.01 ± 6.6	74.56 ± 6.89	74.99 ± 4.95	74.72 ± 6.13	75.4 ± 6.69	56.35 ± 5.06	54.75 ± 5.24	50.75 ± 1.42	50.49 ± 1.14	73.16 ± 5.31	58.43 ± 4.3	66.65
Proteomic signature	74.43 ± 9.36	83.45 ± 5.82	79.65 ± 5.3	79.11 ± 4.92	88.55 ± 2.99	76.91 ± 4.84	87.31 ± 4.46	84.55 ± 5.56	86.23 ± 5.06	86.9 ± 4.08	84.98 ± 4.33	86.01 ± 4.37	79.42 ± 7.54	80.37 ± 4.97	71.32 ± 5.62	54.95 ± 3.51	81.13 ± 4.17	71.22 ± 3.92	79.81
Metabolomic signature	63.9 ± 5.29	80.52 ± 5.3	78.41 ± 5.84	73.38 ± 4.01	79.23 ± 4.77	71.62 ± 3.93	82.15 ± 4.24	78.53 ± 4.0	79.67 ± 4.64	80.47 ± 5.59	78.48 ± 3.57	78.7 ± 6.34	67.66 ± 4.2	68.91 ± 4.17	57.86 ± 5.12	53.9 ± 2.42	57.1 ± 3.57	65.25 ± 4.27	71.98
All Proteins	69.37 ± 6.09	83.21 ± 5.25	77.94 ± 7.5	74.19 ± 6.62	85.96 ± 6.1	76.59 ± 6.14	85.46 ± 5.51	83.11 ± 5.54	79.58 ± 6.36	80.73 ± 6.65	84.12 ± 6.33	84.44 ± 3.72	84.21 ± 3.31	82.7 ± 7.5	73.92 ± 4.57	50.0 ± 0.0	78.6 ± 5.17	63.84 ± 5.41	77.67
All Metabolites	64.59 ± 5.63	75.26 ± 5.23	76.53 ± 5.86	65.47 ± 5.91	76.4 ± 6.18	62.69 ± 6.16	78.75 ± 6.6	74.58 ± 5.75	69.45 ± 6.25	71.75 ± 6.12	73.1 ± 6.33	76.52 ± 7.65	73.0 ± 3.56	71.71 ± 5.15	62.23 ± 5.89	54.64 ± 2.3	58.75 ± 2.46	59.45 ± 4.79	69.16
Proteomics Union (volcano & signature)	77.41 ± 4.2	83.62 ± 5.47	79.48 ± 4.43	75.08 ± 5.84	86.45 ± 4.6	77.44 ± 4.69	87.56 ± 5.1	83.71 ± 5.74	85.5 ± 4.25	85.22 ± 2.98	85.71 ± 4.09	85.65 ± 4.54	78.82 ± 4.68	81.76 ± 4.41	65.87 ± 4.2	51.88 ± 1.8	79.97 ± 4.26	63.16 ± 5.64	78.57
Metabolomics Union (volcano & signature)	68.08 ± 7.54	79.95 ± 4.45	74.75 ± 8.14	69.3 ± 5.89	80.64 ± 4.74	72.9 ± 5.75	81.81 ± 5.89	73.08 ± 6.43	76.91 ± 6.58	79.68 ± 4.06	78.32 ± 5.88	78.24 ± 6.12	70.31 ± 7.24	67.52 ± 6.79	55.93 ± 6.9	53.73 ± 2.61	58.45 ± 5.11	59.08 ± 3.66	71.04
Proteomics volcano plot	72.51 ± 5.26	81.14 ± 7.44	79.9 ± 2.44	73.33 ± 4.38	83.0 ± 5.47	72.94 ± 5.05	83.06 ± 5.29	82.62 ± 2.96	82.75 ± 4.84	84.66 ± 2.73	82.21 ± 6.15	81.9 ± 7.03	76.5 ± 7.7	74.35 ± 5.74	59.85 ± 4.21	52.05 ± 1.79	80.05 ± 4.21	65.28 ± 4.31	76.01
Metabolomics volcano plot	62.16 ± 5.14	71.44 ± 4.89	59.5 ± 4.16	64.66 ± 5.93	77.39 ± 4.21	65.1 ± 5.67	76.17 ± 5.62	64.76 ± 6.09	71.27 ± 4.47	76.4 ± 3.85	72.2 ± 4.78	73.06 ± 7.3	64.76 ± 7.11	65.03 ± 6.65	51.69 ± 2.08	54.92 ± 3.44	58.65 ± 5.11	56.22 ± 6.03	65.85

Figure S3. Balanced accuracy for all the models fitted on tall the versions of the dataset. Rows are the versions of the dataset, columns are the models.

2 SUPPLEMENTARY INFORMATION

2.1 Interpretability in Machine Learning

Interpretability and explainability are notions that are currently at the center of a large number of debates. Therefore, any discussion about those concepts is very interesting. In this work, we mainly rely on the work of Rudin et al. (2021), and Molnar (2022) and hence we consider that interpretability can be seen as a multi-dimensional space, containing approaches with varying

- **Sparsity**: The number of features on which the model relies is a mandatory criterion for the interpretation of its decision.
- **Decision simplicity**: The complexity of the decision function. Even if a decision function relies on, for example, four decision stumps, combining those with logical functions, or weighted majority votes spans a wide spectrum of different function complexities.
- **Learning transparency**: The learning process is similarly important in the interpretation of the decision function. Algorithms such as decision tree are easy to understand in essence, but understanding the Gini score requires a sound mathematical background.

Those characteristics are examples of features on which interpretability relies, and justify why we consider RF and boosting partially interpretable. Indeed, in our case, Random Forest (RF) and Boosting have the great advantage of naturally proposing a quantification of the importance of each feature that have been included in their algorithm. If one considers explainability as the range of post-hoc methods used to understand classifiers, Random Forests and Boosting do not require such methods. Random forests are problematic in the sense that hey output very dense decision functions. However, they have the great advantage of relying on a uniform majority vote, which is much simpler than the linear combination on which boosting relies. In contrast, boosting approaches are usually sparser than Random Forests. Therefore, in this paper, we consider that Boosting and Random Forests are both partially interpretable, as they provide better-than-post-hoc methods to understand their decision, but they still output either dense or mathematically complex decision functions.

REFERENCES

Molnar, C. (2022). *Interpretable Machine Learning*. 2 edn.

Rudin, C., Chen, C., Chen, Z., Huang, H., Semenova, L., and Zhong, C. (2021). Interpretable machine learning: Fundamental principles and 10 grand challenges. *CoRR* abs/2103.11251
